# Correction to: Multi-omic integration by machine learning (MIMaL)

**DOI:** 10.1093/bioinformatics/btad146

**Published:** 2023-04-13

This is a correction to: Quinn Dickinson, Andreas Aufschnaiter, Martin Ott, Jesse G Meyer, Multi-omic integration by machine learning (MIMaL), *Bioinformatics*, Volume 38, Issue 21, 1 November 2022, Pages 4908–4918, https://doi.org/10.1093/bioinformatics/btac631

In the originally published version of this manuscript, in the **Introduction** section, text was missing from the beginning of the second paragraph. This should read: “Machine learning is a promising approach for discovering relationships between datasets. Machine learning techniques have enabled successful integration of multi-omic datasets (Kim *et al.*, 2016)[…]” instead of: “Chai (2021), cellular state in Escherichia coli (Kim *et al.*,2016)[…]”. The publisher apologizes for this error.

This emendation has been made in the article.

